# Non-fatal outcomes of COVID-19 disease in pediatric organ transplantation associates with down-regulation of senescence pathways

**DOI:** 10.1038/s41598-024-52456-y

**Published:** 2024-01-22

**Authors:** Kumar Subramanian, Rency Varghese, Molly Pochedly, Vinona Muralidaran, Nada Yazigi, Stuart Kaufman, Khalid Khan, Bernadette Vitola, Alexander Kroemer, Thomas Fishbein, Habtom Ressom, Udeme D. Ekong

**Affiliations:** 1 Medstar Georgetown Transplant Institute, Medstar Georgetown University Hospital, 3800 Reservoir Rd, NW, Washington, DC USA; 2 Department of Oncology, Genomics, and Epigenomics Shared Resource, Lombardi Comprehensive Cancer Center, Georgetown University Medical Center, Washington, DC USA

**Keywords:** Viral infection, Transplant immunology

## Abstract

This is a cross-sectional study examining kinetics and durability of immune response in children with solid organ transplants (SOTs) who had COVID-19 disease between November 2020 through June 2022, who were followed for 60-days at a single transplant center. Blood was collected between 1–14 (acute infection), and 15–60 days of a positive PCR (convalescence). SOT children with peripheral blood mononuclear cells (PBMC) cryopreserved before 2019 were non-infected controls (ctrls). PBMCs stimulated with 15-mer peptides from spike protein and anti-CD49d/anti-CD28. Testing done included mass cytometry, mi-RNA sequencing with confirmatory qPCR. 38 children formed the study cohort, 10 in the acute phase and 8 in the convalescence phase. 20 subjects were non-infected controls. Two subjects had severe disease. Subjects in the acute and convalescent phases were different subjects. The median age and tacrolimus level at blood draw was not significantly different. There was no death, and no subject was lost to follow-up. During acute infection CD57 expression was low in NKT, Th17 effector memory, memory Treg, CD4^−^CD8^−^, and γδT cells (*p* = 0.01, *p* = 0.04, *p* = 0.03, *p* = 0.03, p = 0.004 respectively). The frequencies of NK and Th2 effector memory cells increased (*p* = 0.01, *p* = 0.02) during acute infection. Non-switched memory B and CD8 central memory cell frequencies were decreased during acute infection (*p* = 0.02; *p* = 0.02), but the decrease in CD8 central memory cells did not persist. CD4^−^CD8^−^ and CD14 monocyte frequencies increased during recovery (*p* = 0.03; *p* = 0.007). Our observations suggest down regulation of CD57 with absence of NK cell contraction protect against death from COVID-19 disease in children with SOTs.

## Introduction

A dysregulated immune response coupled with marked T cell exhaustion and senescence has been postulated to drive severe COVID-19 disease symptomatology and post-acute COVID syndrome (long COVID)^1,2^. So, coordinated resolution of inflammation is necessary for successful recovery following acute infection, and essential to prevent a persistent inflammatory state and dysregulated immune responses^3–5^. While some observational studies have described a mild clinical course of COVID-19 disease in immunosuppressed children with SOT^6,7^, the long-term impact of SARS-CoV-2 infection in this patient cohort remains understudied. Given our observation of successful recovery from mild and severe COVID-19 disease in children with SOT^8^, we sought to study the immune landscape during acute infection and following recovery from COVID-19 disease in children with SOTs. Our objective was to examine the kinetics of immune cell populations during COVID-19 disease and assess how long the changes lasted in response to infection. We also sought to identify markers associated with COVID-19 disease symptom severity in our patient cohort.

## Results

### Participants/descriptive data

38 children were included in this study: 10 had blood collected during the acute phase of infection, 8 had blood collected during the convalescent phase of infection, 20 were solid organ transplanted children who had blood collected during routine transplant care prior to 2019 (non-infected controls). Detailed information of the study subjects is presented in Table 1. 2 patients had severe disease and required supplemental oxygen support but no BIPAP or mechanical ventilation. Neither required cardiovascular support with pressors or Ecmo. Duration of hospitalization was for 18 and 14-days respectively. Of the other patients, 7 were asymptomatic and 9 were symptomatic. Symptoms included fever, cough, congestion, anorexia, vomiting, diarrhea, headache, body ache, and abdominal pain. Tacrolimus was not discontinued. Mycophenolate was held in hospitalized patients who were receiving mycophenolate as supplemental immunosuppression. There were no deaths from COVID-19 disease in our cohort. No patient was lost to follow-up. None of the children were vaccinated against SARS-CoV-2.

**Table 1:** Demographics.

Variable	Acute infection (n = 10)	Convalescent (n = 8)	Non-infected controls (n = 20)	*p* value
Age at blood draw (years)				
Median (IQR)	5.5 (2.92–12.74)	6.7 (3.05–10.08)	2.3 (1.27–6.76)	0.22
Duration from TX at blood draw (years)				
Median (IQR)	3.2 (1.25–5.89)	4.3 (0.71–8.00)	0.52 (0.08–0.98)	0.05
Tacrolimus level at blood draw (ng/dl)				
Median (IQR)	10.1 (6.30–14.40)	6.8 (4.22–8.20)	6.5 (3.65–10.48)	0.16
Sex				
Male	4	5	13	0.46
Female	6	3	7	
Transplant type				
Isolated Liver	7	4	20	**0.02**
Liver/Small bowel	3	4		
Co-morbidities				
0	4	2		0.25
1	3	4		
≥ 2	3	2		

Key.

IQR: 25–75% interquartile range.

TX: transplant.

Significant values are in [bold].

###  Downregulation of senescence with a trend towards upregulation of cytolytic effectors are predominant signatures of COVID-19 disease in children with SOT

**Figure 1:**
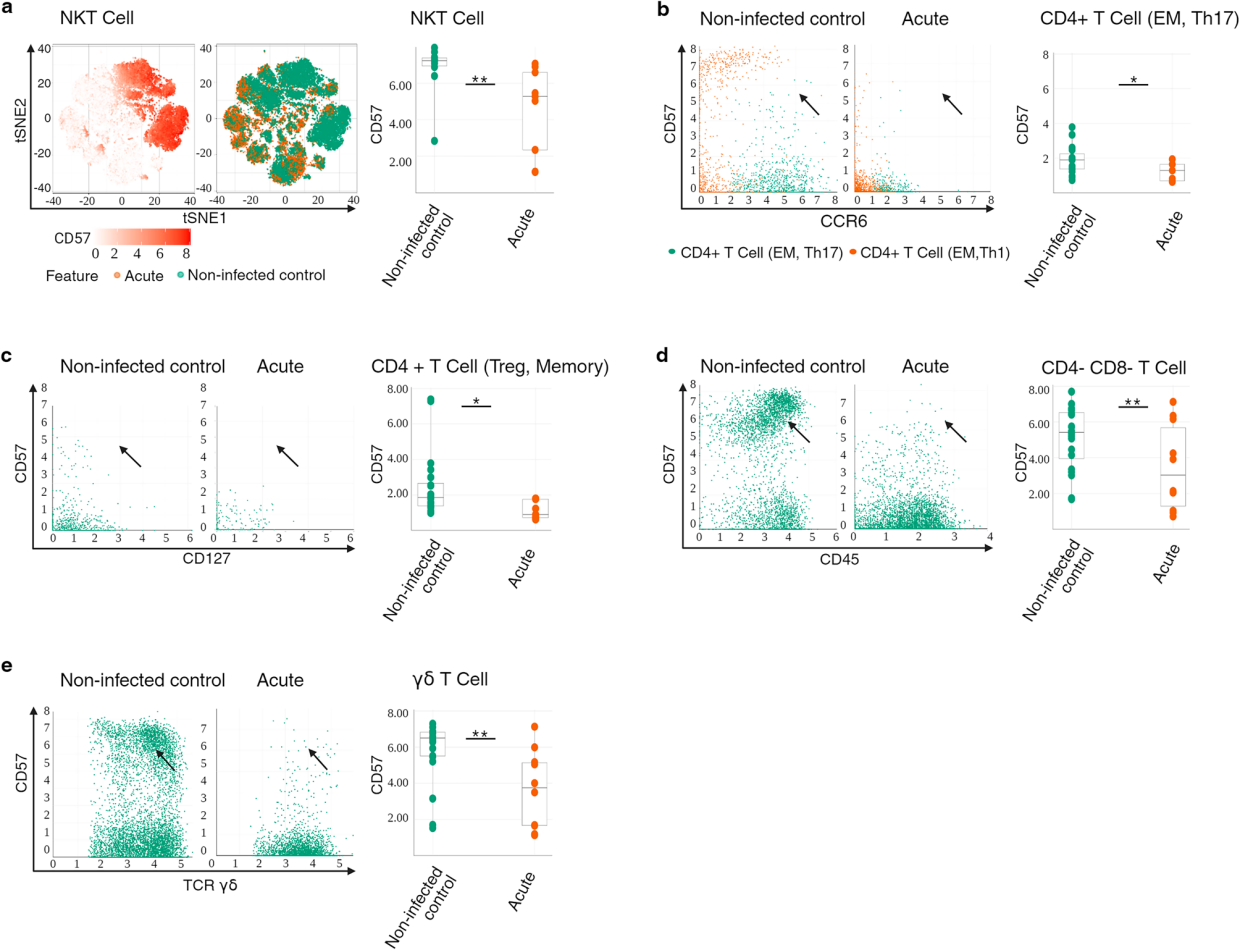
Immunophenotype of peripheral T and NK cell compartments correlates with downregulation of senescence in acute infection. (**A**) t-SNE representation of flow cytometry data comparing NKT cells of patients with acute infection (brown) and NKT cells of non-infected controls (green). Expression levels of CD57 are indicated (white: low expression; deep orange: high expression). Box plots showing summary data. The line in the middle of the box plot represents the median (**B**) Bivariate plots showing frequencies of CD57 within CD4^+^ T cells (EM, Th17) in acute infection and in non-infected controls. The arrowheads pointing to the CD4^+^ T cells (EM, Th17) cell population. Box plots showing summary data. (**C**) Bivariate plots showing frequencies of CD57 within CD4^+^ T cell (Treg, memory) in acute infection and in non-infected controls. The arrowheads pointing to the CD4^+^ T cell (Treg, memory) population. Box plots showing summary data. (**D**) Bivariate plots showing frequencies of CD57 within CD4^−^CD8^−^ T cells in acute infection and in non-infected controls. Arrowheads pointing to the CD4^−^CD8^−^ T cells with high CD57 expression. Box plots showing summary data. (**E**) Bivariate plots showing frequencies of CD57 within γδT cells in acute infection and in non-infected controls. Arrowheads pointing to γδT cells with high CD57 expression. Box plots showing summary data.*p < 0.05 **p < 0.01.

To broadly identify changes occurring during acute infection, the expression of senescence CD57 marker, cytotoxic CD161 marker, co-stimulation CD28 marker, CD66b marker, cytokine and chemokine receptor CD294 and CD185_CXCR5 marker on B cells, granulocytes, T cells, NK cells, and myeloid cells was assessed in acute infected patients vs. non-infected controls. We observed an increased expression of CD161 in the T cell compartment, increased CD66b expression in the myeloid compartment; and decreased expression of CD57 in the T cell compartment (Fig. S1a). Looking at the T cell compartment in more detail, CD57 expression was significantly lower in NKT cells, CD4^+^T cell (EM, Th17), CD4^+^T cell (Treg, memory), CD4^−^CD8^−^ T cell, and γδT cells (Fig. 1a–e) (*p* = 0.01, *p* = 0.04, *p* = 0.03, *p* = 0.03, *p* = 0.004 respectively), and trended downwards in CD4^+^T cell (EM,Th1) (Fig. S1e) (*p* = 0.07). CD161 expression trended in the upward direction in CD4^+^CD8^+^ T cells (Fig. S1f) (*p* = 0.07).

Taken together, these findings suggest that phenotypic characteristics of NKT and CD4^+^T cell compartments in COVID-19 disease in children with SOT is compatible with down-regulation of a marker of senescence, and a trend towards up-regulation of cytolytic effectors in CD4^+^CD8^+^T cells that are primarily of anti-viral specificity.

To assess changes in the immune landscape following recovery from infection, we studied immune cell populations during convalescence compared to acute infection. While the intensity of CD66b expression within the granulocyte and myeloid cell compartments and CD57 expression in the NK cell compartment was increased (Fig. S2a heatmap) during convalescence, the variation was minimal (Fig. S2b,c).

To assess immune system remodeling during COVID-19 disease of different severity, we compared patients with severe vs. mild COVID-19 disease and found increased CD66b in the myeloid and NK cell compartments, and decreased CD57 expression in the T cell compartment in severe disease (Fig. S3a).

**Figure 2:**
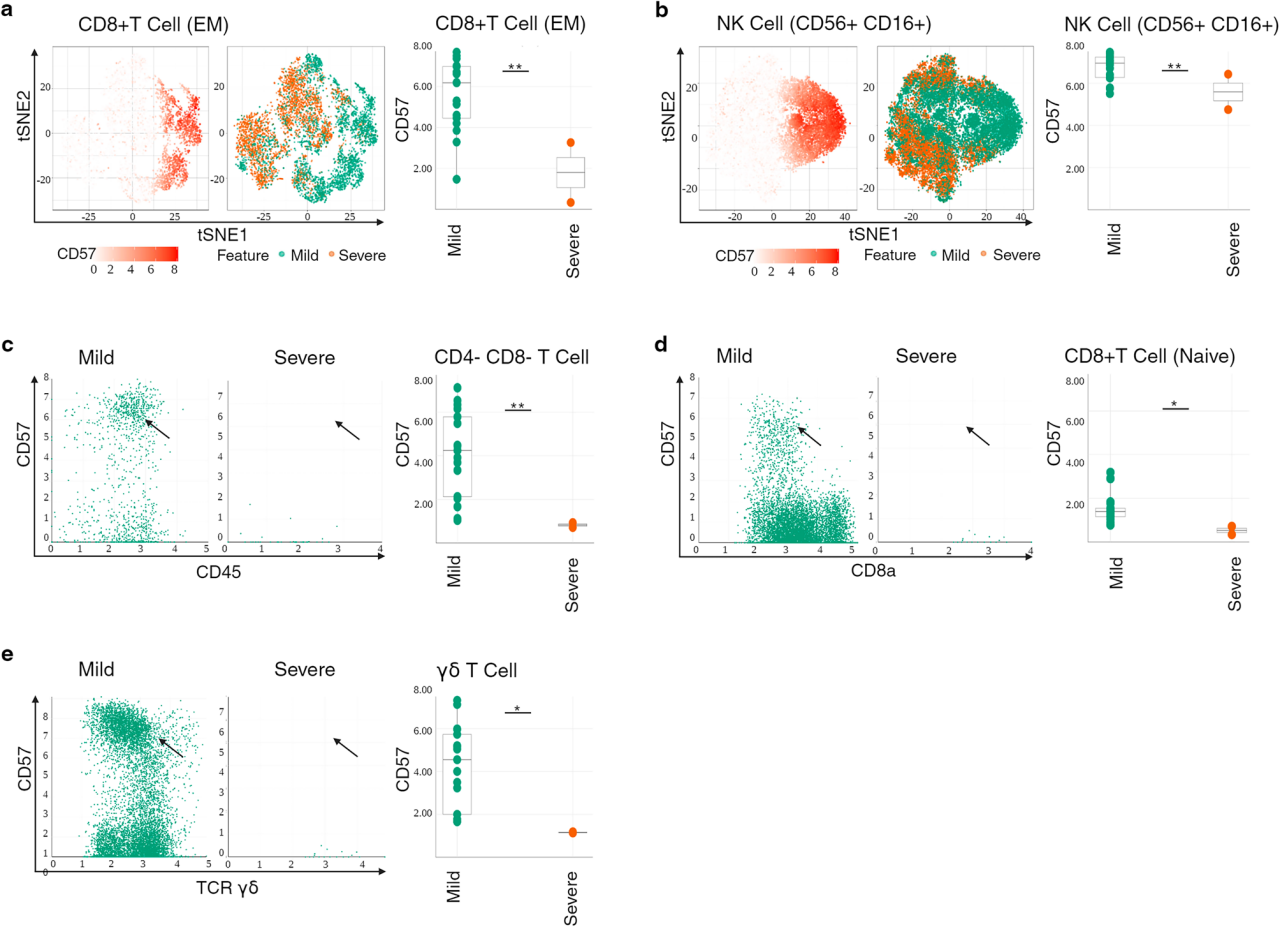
Immunophenotype of peripheral T and NK cell compartments correlates with downregulation of senescence in severe disease. (**A**) t-SNE representation of flow cytometry data comparing CD8^+^T cells (EM) of patients with severe disease (brown) and CD8^+^T cells (EM) of patients with mild disease (green). Expression levels of CD57 are indicated (white: low expression; deep orange: high expression). Box plots showing summary data. The line in the middle of the box plot represents the median. (**B**) t-SNE representation of flow cytometry data comparing NK cell (CD56^+^CD16^+^) of patients with severe disease (brown) and NK cell (CD56^+^CD16^+^) of patients with mild disease (green). Expression levels of CD57 are indicated (white: low expression; deep orange: high expression). Box plots showing summary data. (**C**) Bivariate plots showing frequencies of CD57 within CD4^−^CD8^−^ T cells in severe disease and in mild disease. The arrowhead pointing to CD4^−^CD8^−^ T cells with high CD57 expression. Box plots showing summary data. (**D**) Bivariate plots showing frequencies of CD57 within CD8^+^ T cell (naïve) in severe disease and in mild disease. The arrowhead pointing to CD8^+^ naïve T cells with high CD57 expression. Box plots showing summary data. (**E**) Bivariate plots showing frequencies of CD57 within γδT cells in severe disease and in mild disease. The arrowhead pointing to γδT cells with high CD57 expression. Box plots showing summary data. *p < 0.05 **p < 0.01.

Looking at the T and NK cell compartment in more detail, CD57 expression was significantly lower in CD8^+^T cell (EM), NK cell (CD56^+^CD16^+^), CD4^−^CD8^−^ T cell, CD8^+^T cell (naïve), and γδT cells in severe disease (Fig. 2a–e) (*p* = 0.006, *p* = 0.02, *p* = 0.02, *p* = 0.04, *p* = 0.03 respectively), with a downward trend in CD4^+^ T cell (EM,Th2) (Fig. S3h) (*p* = 0.09).

Altogether our results show that phenotypic characteristics of NK and CD8^+^T cell compartments in children with SOT who have severe COVID-19 disease is compatible with down-regulation of a marker of senescence. Given that antigen-specific T cell responses are critical for viral clearance, and impairment of effector T cell responses is associated with overexpression of inhibitory and senescence pathways^9^, our observation suggests a plausible mechanism that accounts for low mortality from COVID-19 disease in children with SOT.

### Prolonged lymphocyte loss is not a hallmark of COVID-19 recovery in peds SOT

**Figure 3:**
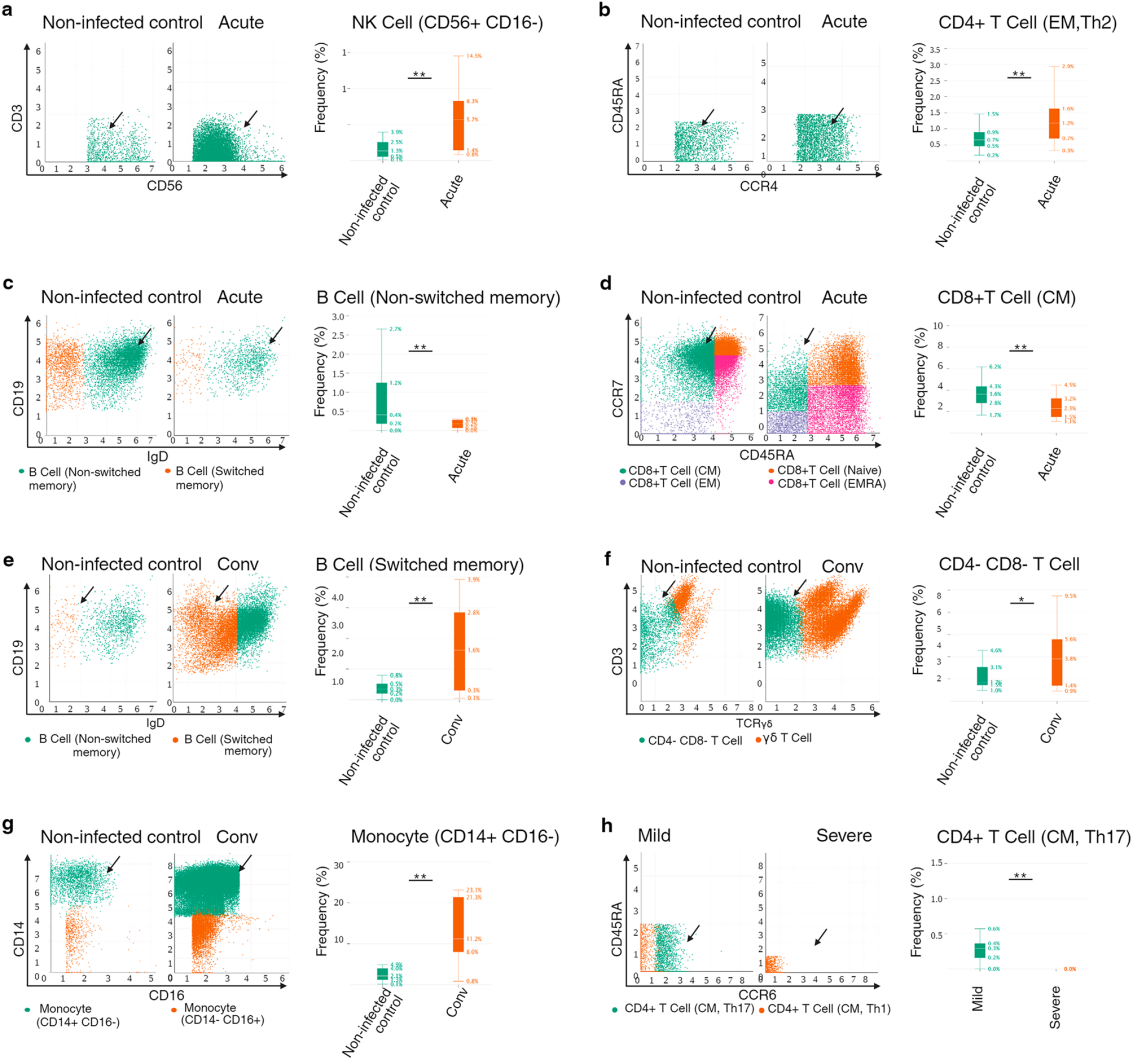
NK cell expansion and imbalance of polarized Th cell subsets characterizes COVID-19 disease in peds SOT. (**A**) Biaxial plot showing expansion of CD56^+^CD16^−^ NK cells during acute infection. Arrowheads pointing to CD16^−^ NK cells. Box plots showing summary data. The line in the middle of the box plot represents the median. (**B**) Biaxial plot showing expansion of CD4^+^ T cell (EM, Th2) during acute infection. Arrowheads pointing to the CD4^+^ T cell (EM, Th2) cell population. Box plots showing summary data. (**C**) Biaxial plot showing decreased frequency of B cell (non-switched memory) during acute infection. Arrowheads pointing to non-switched memory B cells. Box plots showing summary data. (**D**) Biaxial plot showing decreased frequency of CD8^+^ T cell (CM) during acute infection. Arrowheads pointing to CD8^+^ central memory cells. Box plots showing summary data. (**E**) Biaxial plot showing expansion of B cells (switched memory) during recovery. Arrowheads pointing to switched memory B cells. Box plots showing summary data. (**F**) Biaxial plot showing increased frequency of CD4^−^CD8^−^ T cells during recovery. Arrowheads pointing to CD4^−^CD8^−^ T cells. Box plots showing summary data. (**G**) Biaxial plot showing increased frequency of CD14^+^CD16^−^ classical monocytes during recovery. Arrowheads pointing to classical monocytes. Box plots showing summary data. (**H**) Biaxial plot showing decreased frequency of CD4^+^T cells (CM, Th17) during severe disease. Arrowheads pointing at CD4^+^T cells (CM, Th17). Box plots showing summary data. *p < 0.05 **p < 0.01.

During acute infection, differential abundance frequencies of CD56^+^CD16^−^ NK cell and CD4^+^T cell (EM,Th2) were significantly increased (Fig. 3a,b) [− log_10_(FDR):1.59.log(FC):1.82] *p* = 0.01;

[− log_10_(FDR):0.66. log(FC):0.88] *p* = 0.02 respectively and those of B cell (non-switched memory) and CD8^+^T cell (CM) were significantly decreased (Figs. 3c,d) [− log_10_(FDR):0.66. log(FC): − 1.89] *p* = 0.02;

[− log_10_(FDR):0.66. log(FC): − 0.78] *p* = 0.02 respectively. The decrease noted in the CD8^+^T cell (CM) frequency did not appear to persist during recovery as no significant differences were seen during convalescence compared to the non-infected control. CD4^+^T cell (EM,Th2) frequency trended downward upon recovery (Fig. S4a) [− log_10_(FDR): 0.26. log(FC):0.99] (*p* = 0.07) as did CD4^+^T cell (central memory) frequency (Fig. S4b) [− log_10_(FDR):0.26. log(FC):0.80] (*p* = 0.09). B cell (switched memory) frequency trended upwards during recovery (Fig. S4c) [− log_10_(FDR):0.26.log(FC): − 1.62] (*p* = 0.069) and was significantly increased compared to the non-infected controls as would be expected Fig. 3e [− log_10_(FDR):1.45. log(FC):2.05] (*p* = 0.001). Significant increases were noted in CD4^−^CD8^−^T cell and CD14^+^CD16^−^ classical monocyte differential abundance frequencies during recovery (Figs. 3f,g) [− log_10_(FDR):0.69. log(FC):0.8] (*p* = 0.03);[− log_10_(FDR):1.07.log(FC):1.67] (*p* = 0.007) respectively.

Lastly, we observed a significant decrease in the differential abundance frequency of CD4^+^T cell (CM,Th17) in subjects with severe disease (Fig. 3h) [− log10(FDR):0.86, log(FC): − 12.21] (*p* = 0.004).

### SARS-CoV-2- regulated microRNA

Several independent studies demonstrate that RSV infection induces changes in miRNA members belonging to the let-7 and miR-30 families^10^; specifically, let-7b, miR-30a, -30b, -30c are upregulated in RSV infected monocyte-derived dendritic cells (moDCs)^11,12^. Given that induction of some miRNAs seems virus-specific^10^, we investigated miRNA induction by SARS-CoV-2. 53 miRNAs were upregulated and 45 were downregulated in plasma of children with SOT with COVID-19 disease (Fig. S5a). 3 miRNAs stood out in children with severe disease: miR-203a-3p [FDR 0.54; FC -3.68; *p* = 0.003]; miR-18a-5p [FDR 0.99; FC 1.97; *p* = 0.02]; and miR-21-5p [FDR 0.99; FC 1.8; *p* = 0.042] (Fig. S5b). Confirmatory qRT-PCR was performed on the miRNAs with the largest fold change, specifically miR-199a-3p, miRNA-221-3p, miRNA-223-3p, miRNA-24-3p, miRNA-183-5p, miRNA-16-5p; as well as the 3 miRNAs differentially regulated in severe disease (i.e. miR-203a-3p, miR-18a-5p, and miR-21-5p). In the infected vs. non-infected control comparison, the miRNAs that were differentially regulated on miRNA sequencing were not significantly different on qRT-PCR (Fig. S5c). miR-203a-3p was differentially regulated in both sequencing (Fig. S5b) and qRT-PCR in children with severe disease (Fig. S5d) (*p* = 0.05), albeit in different directions. Myd88 is a known target of miR-203a, and miR-203a modulates NfκB signaling in macrophages^13^, in addition to negatively regulating inflammation^14^. Host miRNA-203a has also been reported to be antagonistic to progression of foot-mouth-virus-disease progression in cloven-hoofed livestock^15^.

## Discussion

Taken together, our results show that pediatric SOT patients with COVID-19 disease do not experience long lasting repression of CD4^+^ and CD8^+^ T cell which contrasts with reports in adults^16^. Increased frequency of Th2 cells with an effector memory phenotype is not associated with fatal outcomes in pediatric SOT patients^17^. Contraction in CD56bright NK cells that is particularly evident in adults with fatal outcomes^18^ is not a feature of COVID-19 disease in pediatric SOT; Moreover, the global characteristics of CD56^+^NK cells in children with SOT is not compatible with a senescence phenotype (Fig. 2b) in contrast to reports of a senescence phenotype in NK cells in adults who succumb to infection^18^. SARS-CoV-2 infection did not repress isotype switching of memory B cells in our pediatric SOT cohort, which may have implications for duration of SARS-CoV-2 specific antibody responses and long-term humoral immunity. Our observation of significantly increased classical monocyte frequency during recovery is interesting given that hyperinflammation reported by several groups during severe disease is a result of uncontrolled proliferation of monocytes that secrete inflammatory cytokines and persists for several weeks following recovery. Imbalance of CM, Th17 cell subset reported in adults with severe COVID-19 disease is also present in pediatric SOT with severe COVID-19 disease. Our findings highlight potential reasons for the predominantly mild disease presentation and favorable outcomes even following severe COVID-19 disease in this patient population. To the best of our knowledge, this is the first report detailing the immune landscape with added microRNA data in COVID-19 disease in children with SOT.

NK cells are known to exert primary control during viral infection and CD4^+^ and CD8^+^T cells are critical for long-term surveillance^19^; Loss of CD56bright NK cells is reported in adults with COVID-19 pneumonia and severe disease; and postulated to be a consequence of recruitment of NK cells into infected tissues^20–24^. Thus, the striking observation of increased NK cell frequency during acute infection in our patients suggests NK cells may play a role in effecting a predominantly mild disease phenotype given the favorable outcomes reported in the pediatric SOT patient population^6,8^. Trafficking of NK cells to the lungs occurs in COVID-19 pneumonia and accounts for the low NK cell numbers in the periphery^25^, thus, one could argue that since most of our cohort had mild disease without pneumonia, our result would be expected. However, our cohort included 2 patients with COVID-19 pneumonia. Moreover, NK cells in our patients with severe disease had significantly low CD57 expression, in contrast to observations in critically ill adults with severe COVID-19 disease^9,26^. Given that CD57 is a marker of replicative senescence^27^ and replicative senescence results in a low proliferative capacity of cells and inability to eradicate infection^28,29^, our observations make us question if down regulation of senescence affecting both NK and T cell compartments (in our patients) during severe COVID-19 leads to efficient clearance of SARS-CoV-2 virus. The possibility that down-regulation of CD57 on NK cells in our cohort is reflective of the young age of the subjects and not necessarily reflective of underlying immunopathogenesis of COVID-19 disease seems unlikely given the age at infection is not significantly different between the infected patients and non-infected controls, yet the non-infected controls demonstrated higher CD57 expression compared to the infected cohort.

Compared to non-infected controls, the phenotypic abnormality of T cells during COVID-19 disease is more apparent in SARS-CoV-2-infected patients and patients with severe disease and is associated with low expression of senescence markers. While impairment of effector T cell responses has been associated with overexpression of senescence markers on T cells^30,31^, it is conceivable that CD161^+^ expressing T cells may play an important role in driving non-fatal outcomes as these cells exhibit specificity to viral antigens and demonstrate high levels of cytotoxicity^32–35^. We acknowledge that the cytotoxic potential of CD161^+^ expressing T cells has not been clearly defined in humans^36–39^, and additional studies are needed that determine if in the case of SARS-CoV-2 infection, viral load is controlled by cytotoxic granzymes and perforin secreted by these cells or anti-viral and protective effect is driven by IFN-γ. It is also important to understand the cascade of signaling events that lead to effector functions of CD161 activation during SARS-CoV-2 infection.

Apart from NK and T cells, there are also NKT cells in the peripheral blood that possess cytotoxic capabilities. NKT cells reportedly contribute to the expansion of CD8^+^T cells and amplification of anti-viral responses following respiratory syncytial virus (RSV) infection^40^. Indeed, NKT cell frequency correlates positively with PaO2/FiO2 ratio in adults with COVID-19 disease suggesting it may be a marker of disease severity^20^. As we did not observe differences in differential abundance cell frequencies of NKT cells in our patients with acute infection vs. non-infected controls, their presence may have also contributed to the non-fatal outcomes in our cohort.

Interestingly, some of the cellular immune effects observed in SARS-CoV-2 infection differ from data reported in other respiratory viral infections; specifically, human CD8^+^ T cells or CD4^+^ T cells effectively and independently control RSV replication in human lung tissue in the absence of an RSV-specific antibody response^41^, and both RSV and human metapneumovirus (hMPV) demonstrate a virus-specific and subset-specific effect on myeloid dendritic cell function (mDC)^42^ not reported in SARS-CoV-2 infection. To the best of our knowledge there are no published reports detailing cellular immune changes following RSV and hMPV in infants and children with solid organ transplants, as such we are unable to comment on differences between non-transplanted children and children with solid organ transplants. With regards to SARS-CoV-2 infection in non-transplanted children, some authors attribute mild COVID-19 disease in them to the absence of decreasing T and B lymphocytes, and low prevalence of co-morbid conditions^43^; However, we observed repression of CD4^+^ and CD8^+^ T lymphocytes in children with SOTs with mild disease during the acute phase, albeit not for a prolonged period. Moreover, mild disease occurred in the presence of co-morbid conditions. Neeland et al. have reported that mild disease in non-transplanted children is characterized by reduced circulating subsets of monocytes and natural killer cells during the acute phase^44^; In children with SOT, we observed increased abundance frequencies of natural killer cells with no difference in monocyte subsets during the acute phase. Moreover, classical monocyte differential abundance frequencies increased during convalescence. Our findings therefore provide further insight into immune mechanisms that may contribute to age-related differences in COVID-19 disease in the context of a solid organ transplant. Notably, none of our patients were vaccinated and one of the indications for testing was a sick close contact indirectly suggesting that mild disease in children with SOTs compared to adults represents differences in host and not the virus.

It is unlikely our observations are a consequence of immunosuppression as the tacrolimus trough level was not significantly different at blood draw between infected subjects and non-infected controls, yet clinically meaningful differences in immune cells were observed between the two patient groups that are most likely attributable to SARS-CoV-2 infection and COVID-19 disease. Additionally, the frequency of co-morbidities was not different in the infected and non-infected cohorts. Finally, the CDC classified the predominant variant in the US as: α-January 2020 – May 2021; δ-June 2021 – December 2021; ο-January 2022—present. Subject enrollment spanned these time periods with no obvious differences observed in our findings suggesting that our observations are applicable even during the omicron-predominant variant.

Last but not the least, confirmation of our miRNA findings in a larger cohort with severe disease is important. miRNA-203a which is reported to be a negative regulator of the host protein Sam68 that interacts with foot-mouth-virus thereby modulating both viral translation and RNA replication^15^, is differentially regulated in immunosuppressed children with severe disease. NF-κB signaling is modulated by miR-203a^45^. TNF is thought to be the central mediator of cytokine storm and its devastating consequences in COVID-19 disease^46^, and NF-κB is the major signal transducer molecule in TNF signaling. As none of our patients with severe disease died following infection, it would be important to confirm that miR-203a negatively regulates NF-κB leading to improved outcomes in severe COVID-19 disease. It is also necessary to examine the effect of this miRNA on proteins important to the life cycle of SARS-CoV-2 and SARS-CoV-2 genome as it may have potential bio-therapeutic applications. This is especially important in immunosuppressed patients who remain vulnerable to severe COVID-19 disease and death.

### Limitations of study

There are several limitations of our study. First, the sample size of infected patients is small. This notwithstanding demonstrable differences that are both clinically meaningful and statistically significant can be seen in the acute infection vs. non-infected cohort. Second, we had only two patients with severe disease so while it is tempting to point out differences that appear clinically meaningful, the small number makes it difficult to make big statements. Third, while not all patients with acute infection had blood obtained during the convalescent period, comparison of immune subsets during convalescence vs. non-infected controls showed no significant differences suggesting the changes observed during acute infection were not persistent or long-lasting. This might have implications for post-acute COVID syndrome and warrants further study. Fourth, the acute and convalescent groups were different subjects, and not the same subjects over two different time points. It is therefore possible that the changes we have observed are due to inherent differences between the subjects. Fifth, we were unable to determine if/how immune subsets correlate with clinical indices of disease such as erythrocyte sedimentation rate, C reactive protein, lactate dehydrogenase level, ferritin, d-dimer as these indices were not obtained in every patient with COVID-19 disease or in the controls. Sixth, we acknowledge infected patients and non-infected controls were not age and sex matched however, no significant differences were observed between the cohorts (Table 1). Last but not the least, we acknowledge our observations are associations not cause and effect. We however believe they form the basis for work on mechanism(s) by which NK, NKT, T cells with low CD57 expression protect against mortality in COVID-19 disease. Moreover, they support prior work suggesting the important role they play in disease outcomes.

## Conclusion

Pediatric SOT patients with COVID-19 disease do not experience long lasting repression of CD4^+^ and CD8^+^T cells. Similarly, down regulation of CD57 on T cells together with the absence of contraction of the NK cell population during acute infection might be important protective mechanisms given our observation of predominantly mild disease and absence of death in our cohort; This warrants further study. Repression of isotype switching of memory B cells is not a feature of SARS-CoV-2 infection in pediatric SOT. As a fair number of pediatric SOT recipients remain unvaccinated (by parental choice) the implications of this finding in terms of protective antibody responses and long-term humoral immunity needs further study in this patient population. Lastly, confirmation of the role of miR-203a in NF-κB and TNF signaling in COVID-19 disease warrants further investigation.

## Materials and methods

### Study design/setting/participants

This was a cross sectional study of children with SOTs who had COVID-19 disease between November 2020 through June 2022, and who were followed for up to 60-days following infection. The indication for testing included symptoms, known exposure to COVID-19, or hospital policy prior to procedures, specifically upper endoscopy and colonoscopy, ileoscopy, or liver biopsy. Inclusion criteria were as follows: a positive SARS-CoV-2 nucleic acid test on a nasopharyngeal sample or bronchoalveolar sample, or a positive rapid antigen test at home or in the hospital. All study participants were followed either in the inpatient or outpatient setting at a single transplant center. Blood was collected between 1 and 14-days of a positive PCR for SARS-CoV-2 (acute infection), and between 15 and 60-days of a positive PCR (convalescence), for Ficoll gradient centrifugation of peripheral blood mononuclear cells (PBMC) and collection of plasma. Solid organ transplanted children with PBMC and plasma cryopreserved prior to 2019 served as non-infected controls in experiments described below. None of the children were vaccinated against COVID-19 disease at time of blood collection.

### Variables/data sources/measurement

COVID-19 disease severity was stratified using WHO criteria as follows: 1, no limitation of activities; 2, limitation of activities; 3, hospitalized no O_2_ therapy; 4, O_2_ by mask or nasal prongs; 5, non-invasive ventilation or high flow O_2_; 6, intubation and mechanical ventilation; 7, ventilation and additional organ support. Severe disease was defined as WHO score ≥ 4. Data extracted from electronic medical records included demographic information, tacrolimus levels at time of first noted positive PCR/rapid antigen test, type of transplant, date of transplant, date of first noted positive PCR/rapid antigen test, presence of comorbidities. Comorbidities recorded included obesity, hypertension, hyperlipidemia, diabetes, and chronic lung disease. Obesity was defined as a body mass index at or greater than the 95th percentile for children of the same age and sex according to the CDC definition: https://www.cdc.gov/obesity/childhood/defining.html. Chronic lung disease was defined as a history of asthma or reactive airway disease. Hypertension was identified by the use of anti-hypertensive drug(s). Diabetes was identified by the use of insulin, and hyperlipidemia identified by the use of lipid-lowering drug(s). Chronic kidney disease was identified from the patient problem list in the electronic medical record.

### Study size

No sample size calculations were performed however in the infected vs. non-infected controls, we believe the sample size is adequate to measure the effect size given the highly significant differences demonstrated.

### Statistical methods

Data was analyzed using the Astrolabe Cytometry Platform which follows a standardized pipeline for population identification and statistics^47^. Cells were clustered using FlowSOM^48^ and labeled using the Ek’Balam algorithm. Results were visualized using the t-SNE dimensionality reduction method^49^ using the default parameters from the Rtsne R package. The frequency of the following cell populations: B cell (switched memory, non-switched memory, plasmablast, CD27-), granulocyte (basophil), myeloid (dendritic cell, monocyte), NK cell (CD56^+^CD16^+^, CD56^+^CD16^−^), T cell (CD4^+^ T cell (CM,Th1), CD4^+^ T cell (CM,Th2), CD4^+^ T cell (CM,Th17), CD4^+^ T cell (EM,Th1), CD4^+^ T cell (EM,Th2), CD4^+^ T cell (EM,Th17), CD4^+^ T cell (naïve), CD4^+^ T cell (EMRA), CD4^+^ T cell (Treg, memory), CD4^+^ T cell (Treg, naïve), CD8^+^ T cell Naïve, CD8^+^ T cell (CM), CD8^+^ T cell (EM), CD8^+^ T cell (EMRA), CD4^+^CD8^+^ T cell, CD4^−^CD8^−^ T cell, γδT, NKT) was analyzed as previously described^47^. The expression of CD28, CD57, CD66b, CD161, CD185_CXCR5, and CD294, in the above immune cell populations was also examined.

For the miRNA data, the quantified miRNA expression was normalized and analyzed using DESeq2 algorithm.

For demographic data, continuous variables were analyzed using the Kruskal–Wallis test. Categorical variables were analyzed using Fisher-Freeman-Halton Exact test. Statistical program used SSPS v.28.

The study was approved by the Georgetown University IRB (Study Number: 2017–0365). All research was performed in accordance with relevant guidelines/regulations. Informed consent and assent when applicable was obtained from all legal guardians and participants.

### Separation of PBMC and plasma

Blood was collected into lithium heparinized tubes and immediately processed. PBMCs were isolated using gradient centrifugation in Ficoll-Paque solution as previously described^6,50,51^. Plasma was collected, aliquoted, and stored at − 80 °C for miR-sequencing and confirmatory qRT-PCR.

### Immune phenotyping by mass cytometry (CyTOF)

PBMCs at 1 × 10^6^ cells/ml were stimulated for 16-h with 15-mer peptides from spike protein ((Miltenyi Biotech, Germany) in the presence of co-stimulation with anti-CD49d and CD28. Following overnight incubation, cells were washed and resuspended in 100 μl of cell staining buffer. Each subject sample was stained with CD45 antibody tagged with a different Cadmium isotope (106Cd, 111 Cd, 114Cd, or 116Cd), incubated for 20-min at room temperature (RT), spun down (300×*g* for 5-min), and washed with Maxpar Cell Staining Buffer (CSB) according to manufacturer’s instruction. Each sample was resuspended in 1 ml and four samples combined into one tube. Combined cells are pelleted and resuspended in 270 ul of Maxpar cell staining buffer. 270 ul of cells are added to the MDIPA tubes which contains a dried antibody pellet and the tube gently vortexed and incubated for 30 min at RT. 3 ml of CSB was then added to the tube, gently vortexed and centrifuged (300×*g* for 5 min). Supernatant was gently aspirated, and the wash step repeated one more time. After the second wash, the supernatant aspirated and cells gently vortexed in the residual volume and 1 ml of freshly prepared formaldehyde solution (1.6% final concentration in Maxpar PBS) was added. This was incubated for 10 min at RT and centrifuged at 800xg for 5 min. Cells were carefully aspirated and resuspended in residual volume. 1 ml of intercalation solution (125 nM Cell-ID Intercalator-Ir in Maxpar Fix and Perm Buffer) was added to the tube and gently vortexed. The samples were incubated at 2–8 °C overnight.

Preparation for acquisition on the Fluidigm Hyperion system (Helios component) was as follows: samples were centrifuged at 800×*g* for 5 min and resuspended in 2 ml Maxpar CSB twice. After centrifugation, cells were resuspended in Maxpar Cell Acquisition Buffer (CAS). A cell count was performed to determine final volume for resuspension. For acquisition, cells were resuspended in CAS containing 0.1xEQ beads, filtered and acquired on the Helios. Data files were debarcoded using FCSExpress 7 to produce separate FCS files for each original subject sample.

Supplemental Table 1 lists the CyTOF Antibody Panels. Supplemental Table 2 lists the labeling strategies for each cell subset. Gating follows a hierarchical gating strategy where cells are assigned to increasingly granular subsets (Supplemental Table 2).

### miRNA isolation and heparinase treatment

miRNA isolation from 200 μL plasma was carried out by an miRneasy Plasma/Serum Kit (Qiagen-217204) according to the manufacturer’s recommendations for isolation of total RNA. For normalization of miRNAs, an exogenous miRNA (Cel-miR-39-3p*,* Qiagen, 219,600) was spiked-in during the first step of RNA isolation. Elution of RNA was performed in 14 μL of nuclease-free H_2_O by centrifugation at 8500×*g* for 1 min at 4 °C. RNA concentration was determined using NanoDrop 1000 spectrophotometer. RNA was then treated with heparinase I to overcome the confounding effect of heparin on sequence and qPCR as described previously^52–54^. Briefly, 10 μL of RNA was added to 0.5 μL of heparinase 1 from Bacteroides (NEB, P0735S), 1 μL RNase inhibitor ((Enzymatics, Y9240L), 2 μL of 10 × Reaction Buffer and incubated at 30 °C for 1 h and 99.9 °C for 1 min.

### miRNA sequencing

5ul of heparinase treated RNA was used to build the miRNA library with QIAseq miRNA library kit (Qiagen-331502, Germantown, MD) according to the manufacturer's protocol. The quality of the miRNA libraries was evaluated by the BioAnalyzer DNA High Sensitivity kit (Agilent, Santa Clara, CA). The concentration of the miRNA libraries was determined by Qubit Fluorometer according to the manufacturer's instructions (Thermo Fisher Scientific, Waltham, MA). The pooled miRNA libraries were sequenced on NextSeq 550 High output (Illumina. 20024906) with 75 bp single read to achieve a minimum of 25 M reads per sample (Illumina, San Diego, CA). The sequenced data was analyzed using GeneGlobe® Data Analysis Center to process the reads. The unique molecular index (UMI) counts were calculated, and primary miRNA mapping was performed. Adapter sequences and low-quality bases were removed. UMI counts were analyzed to calculate changes in miRNA expression.

### miRNA expression validation by qRT-PCR

Reverse transcription of miRNAs was carried out using 40 ng of total RNA and the TaqMan™ Fast Advanced master mix Kit (Thermo Scientific-4444557) according to the manufacturer’s recommendations. Real-time PCR was performed using 2 µl of diluted cDNA and TaqMan™ Advanced miRNA cDNA Synthesis Kit (Thermo Fisher Scientific- A28007) Kit following the manufacturer’s guidelines. A run thermal cycling program was utilized in StepOne Plus instrument. The miRNA expression was normalized to cel-miRNA-54 and calculated using the 2^−^*ΔΔ*CT method, where CT is the cycle threshold.

### Supplementary Information

Supplementary Figure S1.

Supplementary Figure S2.

Supplementary Figure S3.

Supplementary Figure S4.

Supplementary Figure S5.

Supplementary Legends.

Supplementary Table S1.

Supplementary Table S2.

## Data Availability

All data is available upon request to the corresponding author, UDE.
