# Supplementary figures and images for: Non-fatal outcomes of COVID-19 disease in pediatric organ transplantation associates with down-regulation of senescence pathways
Source: Sci Rep. 2024 Jan 22;14:1877. doi: 10.1038/s41598-024-52456-y (PMC10803774; doi:10.1038/s41598-024-52456-y)

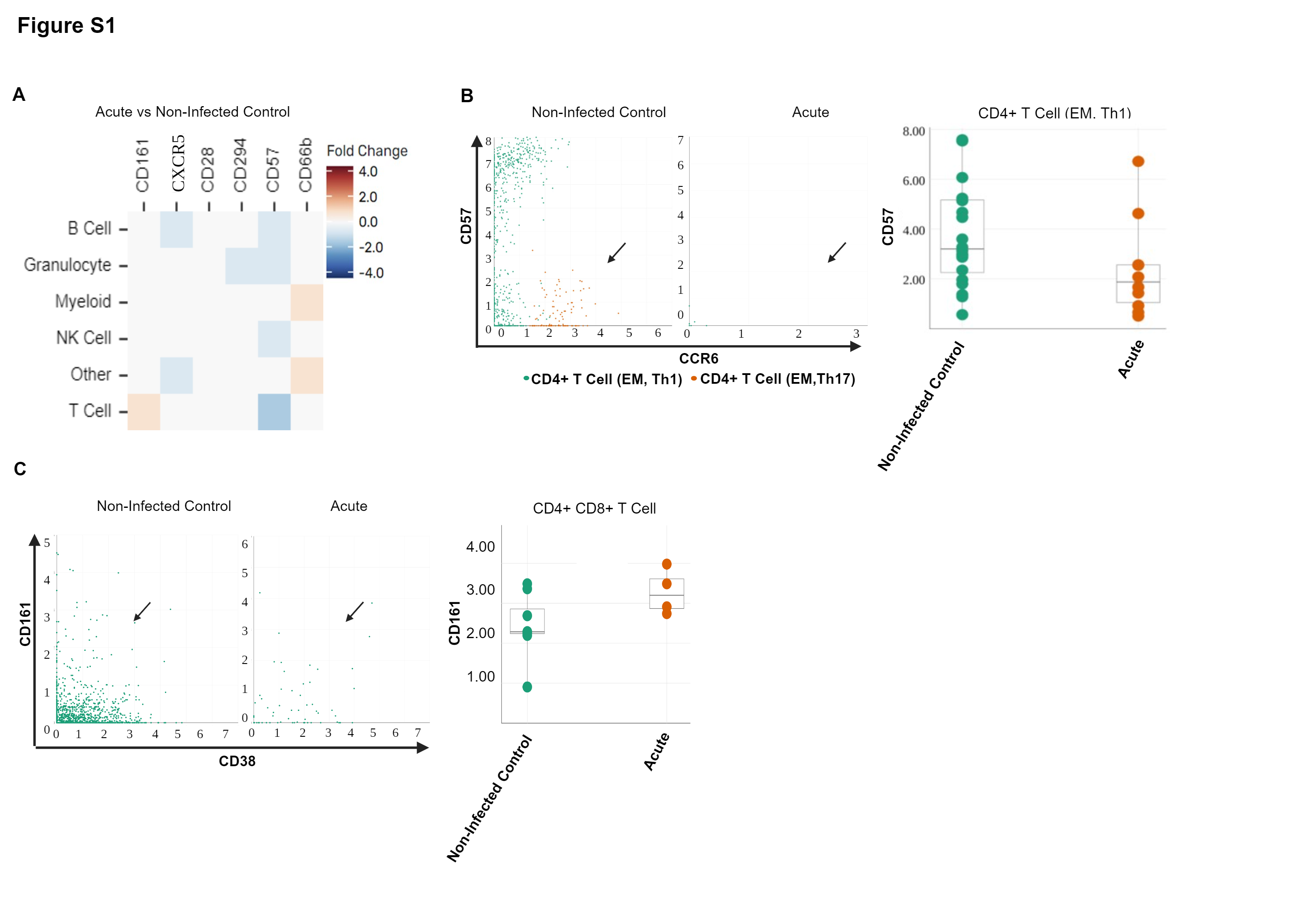

Supplement: Supplementary file 1 — Supplementary Figure S1. [file 41598_2024_52456_MOESM1_ESM.tiff]

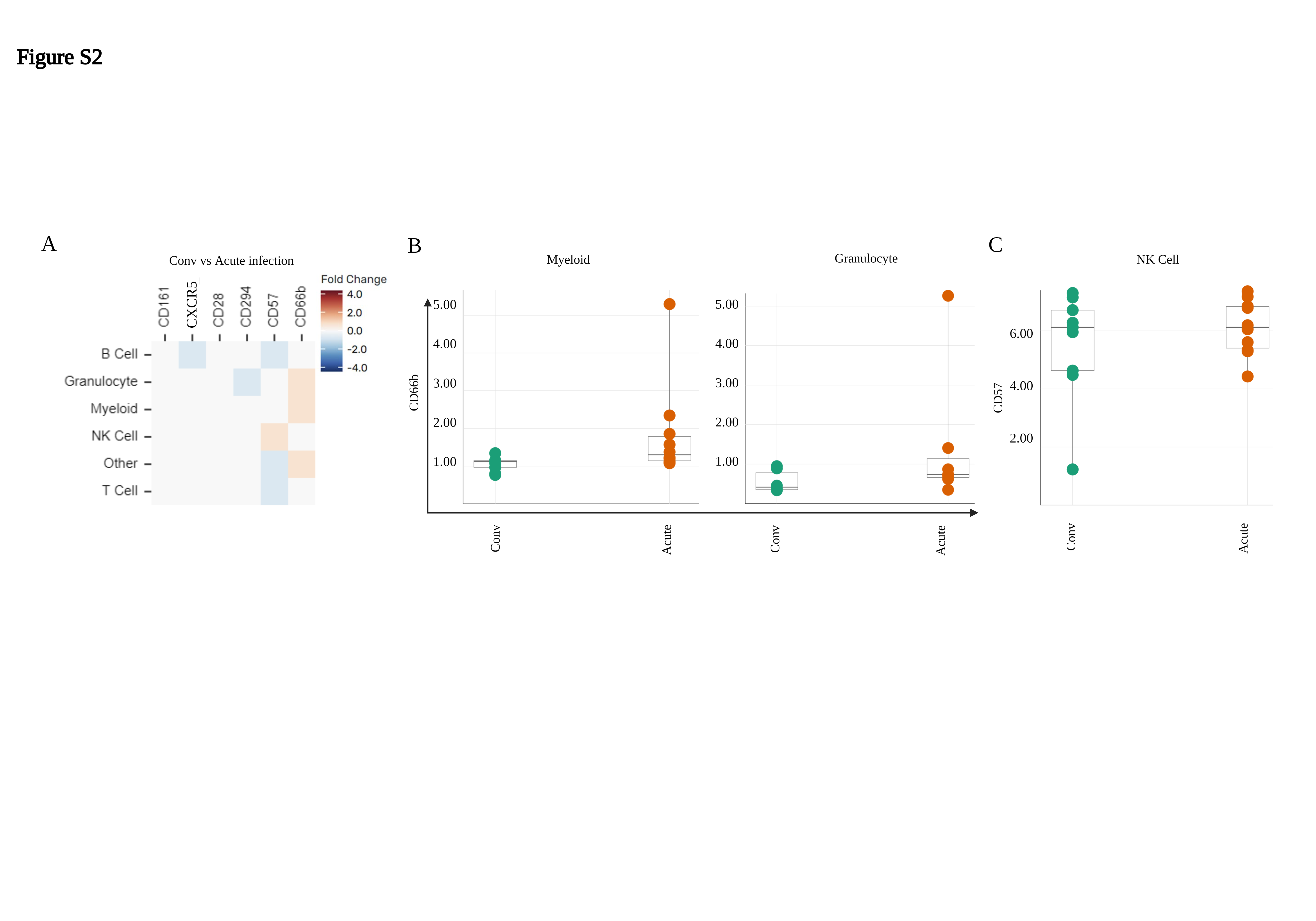

Supplement: Supplementary file 2 — Supplementary Figure S2. [file 41598_2024_52456_MOESM2_ESM.tiff]

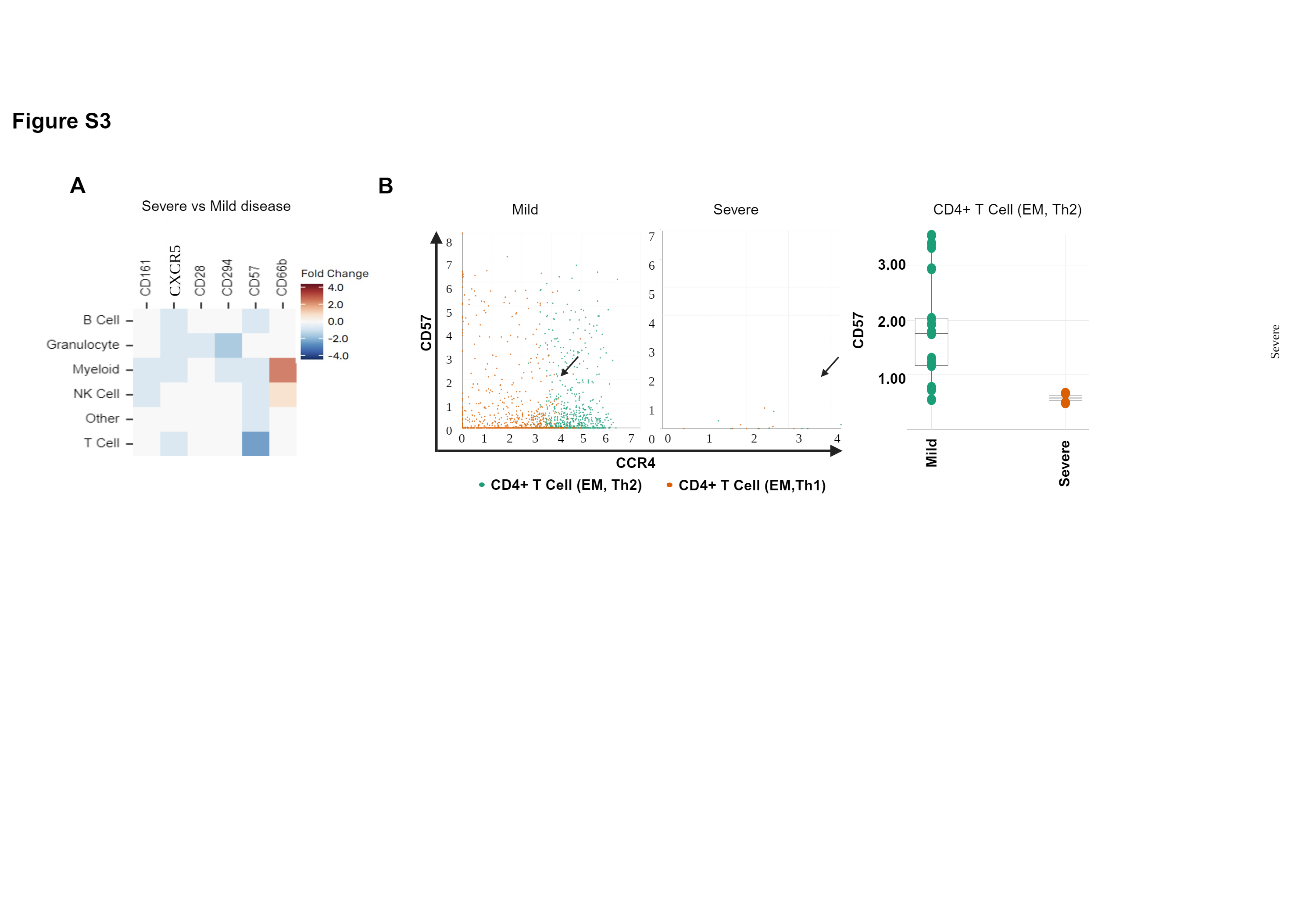

Supplement: Supplementary file 3 — Supplementary Figure S3. [file 41598_2024_52456_MOESM3_ESM.tiff]

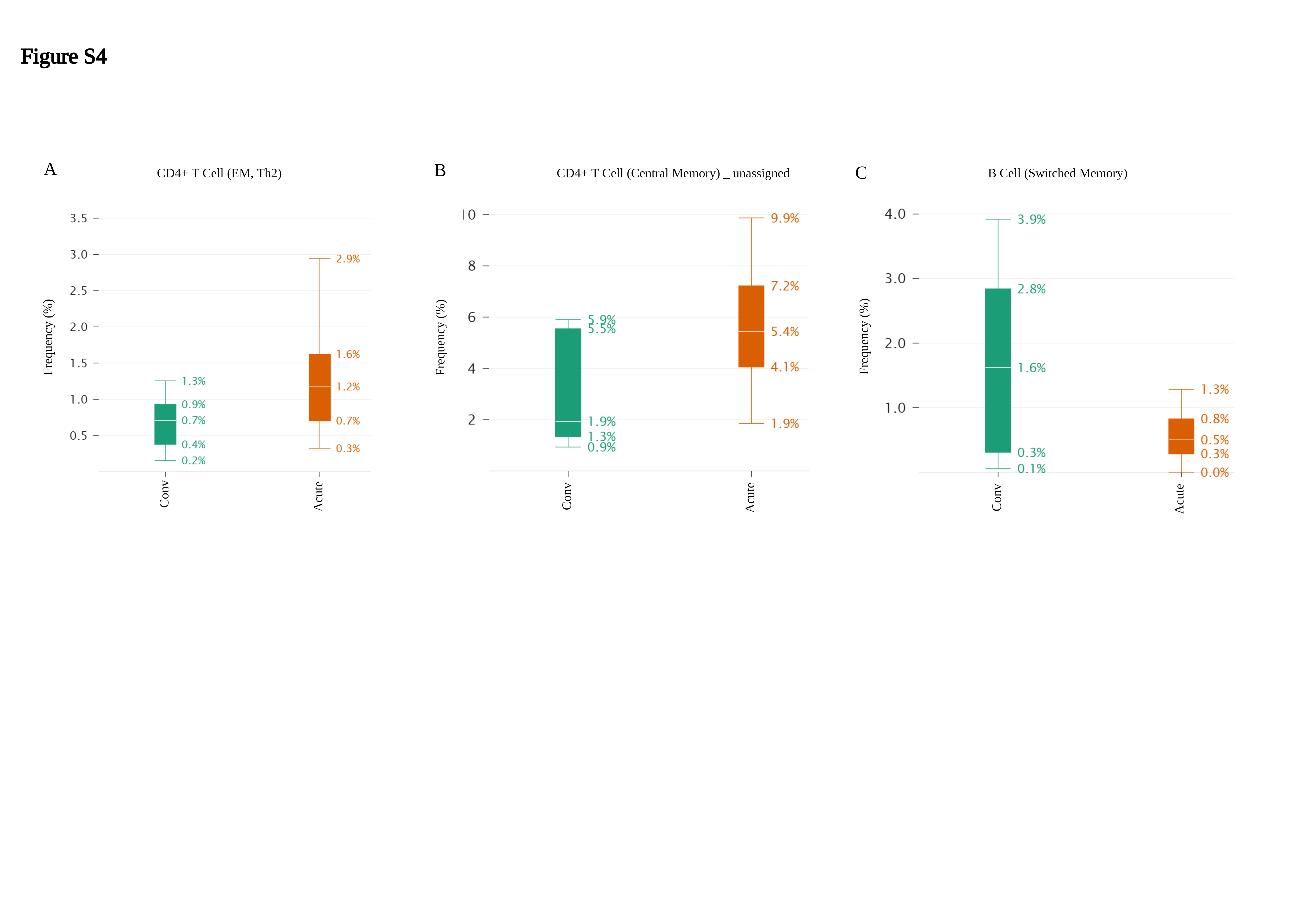

Supplement: Supplementary file 4 — Supplementary Figure S4. [file 41598_2024_52456_MOESM4_ESM.tiff]

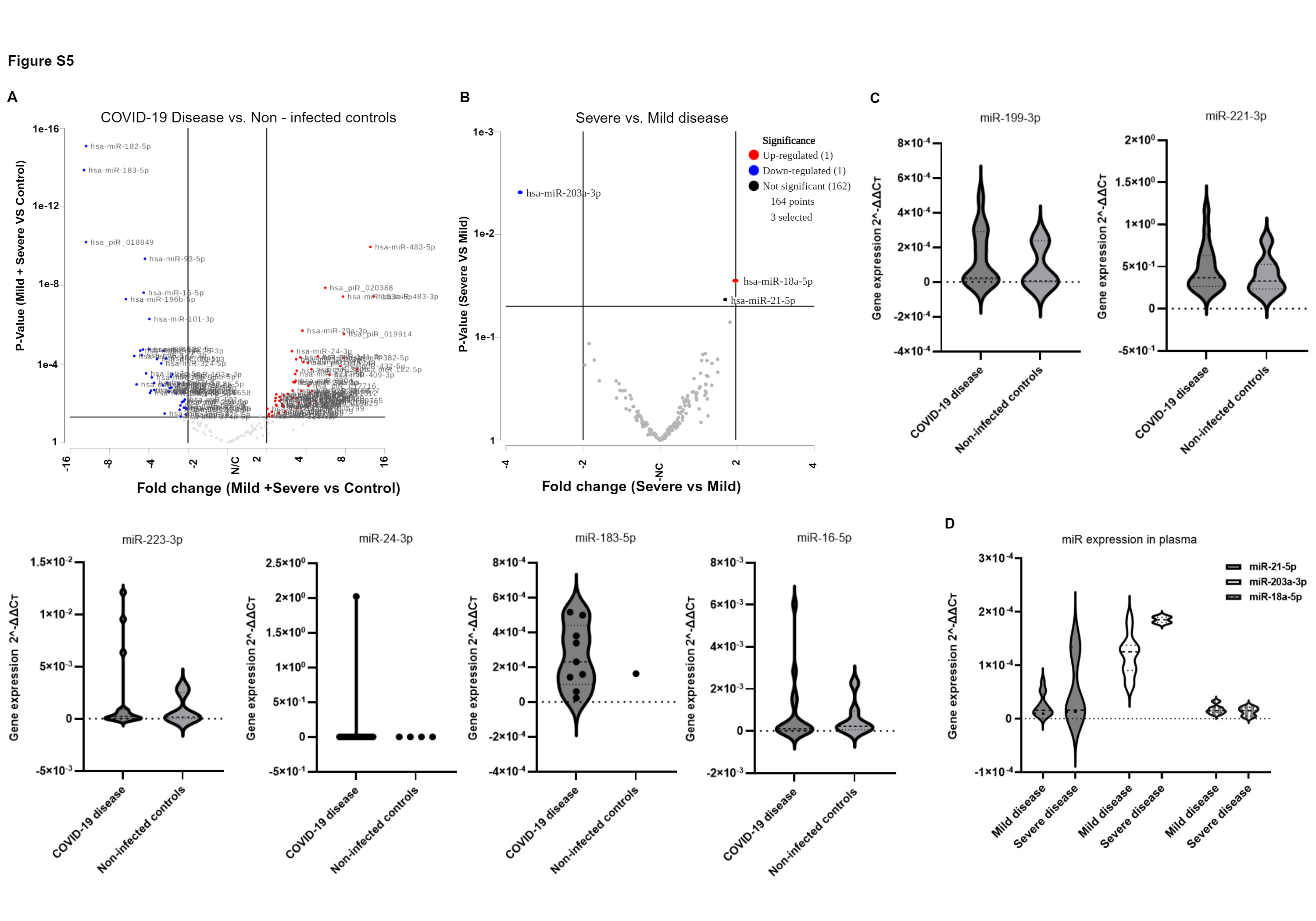

Supplement: Supplementary file 5 — Supplementary Figure S5. [file 41598_2024_52456_MOESM5_ESM.png]
