# Supplementary material for: Non-fatal outcomes of COVID-19 disease in pediatric organ transplantation associates with down-regulation of senescence pathways
Source: Sci Rep. 2024 Jan 22;14:1877. doi: 10.1038/s41598-024-52456-y (PMC10803774; doi:10.1038/s41598-024-52456-y)
Supplement: Supplementary file 6 — Supplementary Legends. [file 41598_2024_52456_MOESM6_ESM.docx]

Supplementary figure legend.

**Figure S1. Differential expression analysis of CyTOF data in acute infection.** a. Surface marker expression profiles of identified immune populations. Markers are on the X axis and immune cell compartments are on the Y axis. Each cell represents the fold change between acute infection and non-infected controls. The more saturated the color, the greater the fold change (blue: low expression; deep orange: high expression). b. Bivariate plots showing frequencies of CD57 within CD4^+^T cells (EM,Th1) in acute infection and in non-infected controls. Box plots showing summary data The line in the middle of the box plot represents the median. c. Bivariate plots showing frequencies of CD161 within CD4^+^CD8^+^ T cells in acute infection and in non-infected controls. Box plots showing summary data.

**Figure S2. Differential expression analysis of CyTOF data during recovery (convalescence).** a. Surface marker expression profiles of identified immune populations. Markers are on the X axis and immune cell compartments are on the Y axis. Each cell represents the fold change between convalescence and acute infection. The more saturated the color, the greater the fold change (blue: low expression; deep orange: high expression). b. CD66b expression within the myeloid and granulocyte compartment during convalescence. c. CD57 expression within the NK cell compartment during convalescence.

**Figure S3. Differential expression analysis of CyTOF data in severe disease.** a. Surface marker expression profiles of identified immune populations. Markers are on the X axis and immune cell compartments are on the Y axis. Each cell represents the fold change between severe disease and mild disease. The more saturated the color, the greater the fold change (blue: low expression; deep orange: high expression). b. Bivariate plots showing frequencies of CD57 within CD4^+^ T cells (EM,Th2) in severe disease and in mild disease. Box plots showing summary data The line in the middle of the box plot represents the median. p=0.09.

**Figure S4. Differential abundance frequencies during recovery (convalescence).** a. CD4^+^T cell (EM,Th2) frequency trended downwards during convalescence {-log_10_(FDR): 0.26. log(FC):0.99 {p=0.07}. b. CD4^+^T cell (central memory) frequency trended downwards during convalescence {-log_10_(FDR):0.26. log(FC):0.80}{p=0.09}. c. B cell (switched memory) frequency trended upwards during convalescence {-log_10_(FDR):0.26.log(FC):-1.62}{p=0.069}.

Box plots showing median and standard deviation for cell frequency. Data obtained from 8 COVID-19 recovered children and 10 COVID-19 acutely infected children.

**Figure S5. Differential microRNA expression.** a. Volcano plot showing comparison of miR expression in COVID-19 disease vs. non-infected controls. 53 miR’s significantly upregulated with >2fold change (red) and 45 miR’s significantly downregulated with >2fold change (blue).

X axis: fold change. Y axis: p value. Horizontal line at p=0.05. Vertical lines at fold change ±2. b. Volcano plot showing comparison of miR expression in severe disease vs. mild disease. miR-203a-3p significantly down-regulated {FDR 0.54; FC -3.68; p=0.003} and miR-18a-5p significantly up-regulated {FDR 0.99; FC 1.97; p=0.02} in severe disease. miR-21-5p also significantly up-regulated {FDR 0.99; FC 1.8; p=0.042}in severe disease.

X axis: fold change. Y axis: p value. Horizontal line at p=0.05. Vertical lines at fold change ±2.

c. Confirmatory qRT-PCR of miRNAs with the largest fold change. Violin plots show miR-199a-3p, miRNA-221-3p, miRNA-223-3p, miRNA-24-3p, miRNA-183-5p, miRNA-16-5p expression in COVID-19 disease vs. non-infected patients.

d. Confirmatory qRT-PCR of significant miRNAs in the mild vs. severe disease comparisons. Violin plots show a trend towards miR-203a-3p upregulation in severe disease (p=0.05).
