# Supplementary material for: Non-fatal outcomes of COVID-19 disease in pediatric organ transplantation associates with down-regulation of senescence pathways
Source: Sci Rep. 2024 Jan 22;14:1877. doi: 10.1038/s41598-024-52456-y (PMC10803774; doi:10.1038/s41598-024-52456-y)
Supplement: Supplementary file 7 — Supplementary Table S1. [file 41598_2024_52456_MOESM7_ESM.docx]

Supplemental Table 1. CyTOF Antibody Panels

| **Surface Staining Panel** | | | |
| --- | --- | --- | --- |
| **Isotope** | **Target** | **Clone** | **Source** |
| Sm147 | CD11c | Bu15 | Fluidigm |
| Nd143 | CD123_IL_3R | 6H6 | Fluidigm |
| Yb176 | CD127_IL_7Ra | A019D5 | Fluidigm |
| Er168 | CD14 | 63D3 | Fluidigm |
| Nd148 | CD16 | 3G8 | Fluidigm |
| Eu151 | CD161 | HP-3G10 | Fluidigm |
| Gd156 | CD183_CXCR3 | G025H7 | Fluidigm |
| Gd158 | CD185_CXCR5 | J252D4 | Fluidigm |
| Nd144 | CD19 | HIB19 | Fluidigm |
| Sm152 | CD194_CCR4 | L291H4 | Fluidigm |
| Pr141 | CD196_CCR6 | G034E3 | Fluidigm |
| Er167 | CD197_CCR7 | G043H7 | Fluidigm |
| Yb171 | CD20 | 2H7 | Fluidigm |
| Eu153 | CD25_IL_2Ra | BC96 | Fluidigm |
| Sm154 | CD27 | O323 | Fluidigm |
| Gd160 | CD28 | CD28.2 | Fluidigm |
| Er166 | CD294 | BM16 | Fluidigm |
| Er170 | CD3 | UCHT1 | Fluidigm |
| Dy161 | CD38 | HB-7 | Fluidigm |
| Nd145 | CD4 | RPA-T4 | Fluidigm |
| Y89 | CD45 | HI30 | Fluidigm |
| Nd150 | CD45RA | HI100 | Fluidigm |
| Sm149 | CD45RO | UCHL1 | Fluidigm |
| Dy163 | CD56_NCAM | NCAM16.2 | Fluidigm |
| Gd155 | CD57 | HCD57 | Fluidigm |
| Yb172 | CD66b | G10F5 | Fluidigm |
| Nd146 | CD8a | RPA-T8 | Fluidigm |
| Yb173 | HLA_DR | LN3 | Fluidigm |
| Yb174 | IgD | IA6-2 | Fluidigm |
| Dy164 | TCRγδ | B1 | Fluidigm |
| Rh103 | Live_Dead | N/A | Fluidigm |
